# Current approaches in treatment of triple-negative breast cancer

**DOI:** 10.7497/j.issn.2095-3941.2015.0030

**Published:** 2015-06

**Authors:** Hanan Ahmed Wahba, Hend Ahmed El-Hadaad

**Affiliations:** Clinical Oncology and Nuclear Medicine, Mansoura University, Mansoura 35516, Egypt

**Keywords:** Breast cancer, triple-negative, HER2

## Abstract

Triple-negative breast cancer (TNBC) is diagnosed more frequently in younger and premenopausal women and is highly prevalent in African American women. TNBC is a term derived from tumors that are characterized by the absence of ER, PgR, and HER2. So patients with TNBC do not benefit from hormonal or trastuzumab-based therapies. TNBCs are biologically aggressive, although some reports suggest that they respond to chemotherapy better than other types of breast cancer, prognosis remains poor. This is due to: shortened disease-free interval in the adjuvant and neoadjuvant setting and a more aggressive course in the metastatic setting.

## Introduction

Breast cancer is the second leading cause of deaths in women after lung cancer and it is the most common cancer among women worldwide (23% of all new cancer cases)^1^. Triple-negative breast cancer (TNBC) refers to the breast cancer phenotype where the estrogen and progesterone receptor are negative, as assessed by immunohistochemistry (IHC) and there is a lack of overexpression of HER2 as assessed by IHC or the absence of its gene amplification as assessed by fluorescence *in situ* hybridization technique^2^.

An estimated 1 million cases of breast cancer are diagnosed annually worldwide. Of these, approximately 170,000 (12%-20%) are of the triple-negative (ER–/PR–/HER2–) phenotype^3^. Of these TNBC cases, about 75% are “basal-like”^4^. As regard the molecular complexity of TNBC, six subtypes of TNBC have been identified, basal-like (BL1 and BL2), an immunomodulatory (IM), a mesenchymal (M), a mesenchymal stem-like (MSL), and a luminal androgen receptor (LAR) subtype. TNBC is an important area of research for both researchers and clinicians because (I) TNBC is a poor prognostic factor for disease-free survival (DFS) and overall survival (OS); (II) no effective specific targeted therapy is readily available for TNBC; (III) there is a clustering of TNBC cases in premenopausal women and in women of African descent; and (IV) the overlap of BRCA1-associated breast cancers with the TNBC phenotype is significant.

## Treatment modalities of TNBC

Patients with TNBC do not benefit from hormonal or trastuzumab-based therapy because of the loss of target receptors such as ER, PGR, and HER-2. Hence, surgery and chemotherapy, individually or in combination, appear to be the only available modalities. However, some studies have identified certain receptors as targets for new therapeutic drugs.

### Surgery in TNBC

#### Effect of TN status on surgical decision making

Many studies are done to determine whether patients with TN disease were more likely to choose mastectomy over lumpectomy. The result was that TN status, while being associated with younger age and higher grade tumors does not impact surgical treatment choice. Despite the fact that TN disease tend to be more aggressive, surgical decision making likely rests on more traditional clinicopathological variables and patient preference^5^. Freedman *et al*.^6^ had concluded that the local recurrence rate after breast conservative surgery (BCS) is not high in TNBC as those of other subtypes of breast cancer so they remain appropriate candidates for breast conservation.

### Radiotherapy in TNBC

#### Effect of TN status on adjuvant radiotherapy

Traditionally radiotherapy is given in TNBC as indicated in other breast cancer subtypes following mastectomy or conservative breast surgery (CBS), but there is still controversy on this issue^7^. As TNBC are rapidly growing and locally aggressive cancers, CBS followed by radiation therapy in early stage (T_1-2_N_0_) may not be equivalent to mastectomy as in other types of breast cancer^8^. However, Abdulkarim *et al*.^9^ reported that women with TNBCs harbor a pathogenic mutation in the BRCA1 gene and tumors lacking functional BRCA1 are deficient in double-strand DNA break repair by homologous recombination and are potentially highly radiosensitive. If CBS is followed by radiotherapy, the breast and surrounding tissue could eradicate occult BRCA1-deficient tumor foci and thereby decrease locoregional recurrence in those patients.

### Chemotherapy in TNBC

TNBC are biologically aggressive. Although some reports suggest that they respond to chemotherapy better than other types of breast cancer, prognosis remains poor^10^. This is due to: shortened disease-free interval in the adjuvant and neoadjuvant setting and a more aggressive course in the metastatic setting.

The therapeutic strategies for the management of TNBC are targeting DNA repair complex like (platinum compounds and taxanes), P53 like (taxanes), cell proliferation like (anthracycline containing regimen) and targeted therapy^11^. Also several neoadjuvant studies have sought to determine the additive benefit of incorporating novel chemotherapeutics with standard chemotherapy like anthracycline, taxanes, antimetabolites, platinum agents and novel microtubule stabilizing agents^12^. Although the specific adjuvant regimens that may be most effective for TNBC remains incompletely defined for both early stage and advanced disease, third-generation chemotherapy regimens using dose dense or metronomic polychemotherapy like those offered to other high-risk patients are among the most effective tools presently available^13^. Platinum agents have seen renewed interest in TNBC since the association of BRCA1 mutations and dysfunctional DNA repair with TN may indicate an increased sensitivity toward DNA-damaging agents like platinum agents based on preclinical and clinical data. Sensitivity has also been shown to DNA double-strand breaks, such as those induced by etoposide and bleomycin^14^.

Whereas patients with HER-2-overexpressing have repeatedly been indicated to derive the most pronounced benefit from anthracycline-containing chemotherapy, results on the efficacy of anthracycline-based regimens in patients with TNBC remain controversial^15^^,^^16^.

Taxanes are active in TNBC and remain important agents but have not shown specific benefit over non-TNBC^17^^,^^18^. The chemosensitivity of tumors harbouring p53 mutations, a characteristic of TNBC is controversial as resistance of p53-mutated breast cancers to anthracycline chemotherapy has been reported^19^. In the metastatic setting, TNBC patients with higher rates of visceral metastases have a relatively shorter median survival of 7-13 months and have limited duration of response to successive lines of chemotherapy. It is important to select the agents most likely to result in a meaningful benefit^20^^,^^21^.

TNBC is itself a heterogeneous group. Therefore, the identification of molecular biomarkers to predict response to specific chemotherapy is required to further improve treatment strategies with the current menu of chemotherapy options and future combinations with targeted therapies^22^.

#### Neoadjuvant chemotherapy in TNBC

The neoadjuvant setting provides a model for rapid assessment of treatment efficacy with smaller patient accruals and over shorter periods of time compared to the traditional adjuvant setting^12^. Neoadjuvant chemotherapy studies have consistently reported higher response rates (RR) in TNBC than non-TNBC and pathologic complete response (pCR) has been shown to predict improved long-term outcomes for TNBC^18^. There are several features inherent to TNBC that have consistently been shown to be associated with clinical and pathological responsiveness to neoadjuvant chemotherapy like ER negativity and high expression of Ki-67. Also, the neoadjuvant setting provides an opportunity to determine *in vivo* tumor responses to chemotherapy^14^. The association of pCR with survival outcomes has also been observed in neoadjuvant studies thus, pCR is now considered to be an important endpoint in clinical trials assessing the efficacy of neoadjuvant chemotherapy^12^.

##### Neoadjuvant anthracycline and taxanes based regimen

Dees *et al*.^23^ had reported clinical and pathological RRs for neoadjuvant anthracycline–cyclophosphamide-based (AC) chemotherapy as being significantly higher in ER- and HER-2-negative patients compared with other subtypes. Despite this, BL and HER-2-positive/ER-negative subtypes experienced a significantly decreased DFS and OS compared with patients with ER-positive luminal subtypes.

Le Tourneau *et al*.^24^ had reported an enhanced RRs to anthracyclines may be achieved by increasing either dose intensity/density of the applied chemotherapy, an increase in pCR rate from 13% to 47% by intensifying conventional neoadjuvant FEC100 chemotherapy to E70C 700 mg/m^2^ (d1+8) in combination with standard 5-FU (d1-5).

In a retrospective study of a number of patients receiving neoadjuvant anthracycline and taxane based therapy, those patients with TNBC (14%) had significantly higher pCR rates compared to non-TNBC (38% *vs*. 12%). Patients who achieved a pCR had a prolonged DFS and among patients who did not achieve a pCR, the TNBC subgroup had a significantly worse prognosis^25^.

The NSABP B-27 trial evaluated the response to neoadjuvant therapy and long term outcomes where patients received either four cycles of standard (AC) every 3 weeks followed by surgery or four cycles of (AC) followed by four cycles of docetaxel (D) and then surgery or four cycles of AC followed by surgery and then four cycles of adjuvant docetaxel. The addition of preoperative docetaxel nearly doubled the pCR rate from 12.9% and 14.4% in each of the two AC arms, to 26.1% in the AC-D arm. However; the addition of docetaxel did not result in improved DFS or OS in this subgroup^26^.

Rouzier *et al*.^27^ had evaluated the effect of preoperative chemotherapy in 22 basal-like breast cancer patients who are treated with 12 weeks of weekly paclitaxel followed by 4 cycles of fluorouracil, doxorubicin, and cyclophosphamide (FAC), the result revealed a pCR rate of 45%.

##### Neoadjuvant platinum agents in TNBC and BRCA mutation

Several groups have demonstrated that tumor cell lines (human breast and ovary) deficient in BRCA1 are unusually sensitive to the DNA cross-linking agents including cisplatin and mitomycin and that this sensitivity is reversed with either BRCA1 up-regulation or restoration of BRCA1 function^28^.

There has been renewed interest in cisplatin for the treatment of TNBC, in part because of improved strategies for managing its side effects and because of additional preclinical data that have suggested that platinum agents may be particularly active in TNBC due to the histological similarities between BRCA1 mutated breast cancer and TNBC^29^. But it is to be noted that while nearly all BRCA1 tumors are basal-like, not all basal-like tumors have BRCA1 mutations^30^. Preoperative therapy with platinum in TNBC has yielded promising results. Preoperative phase II study conducted by Garber *et al*.^31^ evaluated single agent cisplatin (75 mg/m^2^) given for 4 cycles to women with stage II or III TNBC. The pCR rate was 22% which is not bad for a single agent and 36% had a Miller-Payne score of 4 or 5 which includes complete and near-complete responses. Seven percent of women in this study were BRCA1 carriers and they achieved a pCR. A preliminary biomarker assessment of p63/p73 expression in all available samples demonstrated that a pCR was achieved in 33% biomarker positive patients but only in 7% biomarker negative patients. Platinum agents have also been used in combination with other agents in the neoadjuvant setting. Ezzat *et al*.^32^ had conducted phase II study of preoperative paclitaxel and cisplatin demonstrated a 28% complete RR and 63% partial RR including patients with ER-ve and HER2-ve status. A remarkably high pCR rate of 65% was seen in a number of patients with TNBC treated with cisplatin 30 mg/m^2^, epirubicin 50 mg/m^2^ and paclitaxel 120 mg/m^2^ weekly for 8 weeks. Adjuvant therapy with 4 cycles of cyclophosphamide, methotrexate, and flurouracil (CMF) was administered to all patients and those with four or more positive nodes after preoperative therapy received an additional 4 cycles. Those patients who achieved a pCR had a 3- and 5-year DFS of 97% and 90% respectively compared with 3- and 5-year DFS rates of 61% and 56% in those with residual disease after preoperative therapy^33^.

##### Neoadjuvant antimetabolites

The NSABP study was performed on a number of patients of whom 41% were TN. The aim of the study is to evaluate the response of adding either capecitabine (X) or gemcitabine (G) to docetaxel (T) followed by AC. No statistically significant difference was observed for pCR in both breast and lymph nodes across all treatment arms: T → AC 26%; TX → AC 23.3%; TG → AC 27.3%^34^.

A second study was done to determine the additional benefit of preoperative capecitabine to docetaxel either sequentially or in combination to treat women with HER2-negative breast cancer. In this study, 41.2% of women had TNBC and were treated with either 4 cycles of docetaxel followed by 4 cycles of capecitabine or 8 cycles of concurrent docetaxel/capecitabine. pCR rates were 8% and 11.5% for Arm A and B, respectively. Among those with TNBC, pCR rate in both arms combined was 19%^35^.

Albain *et al*.^36^ had treated women with advanced breast cancer using paclitaxel 175 mg/m^2^ or gemcitabine 1,250 mg/m^2^ on days 1 and 8 plus paclitaxel 175 mg/m^2^ on day one every 3 weeks. The primary end-point of the study was OS. The addition of gemcitabine to paclitaxel resulted in increased RR (40.8% *vs*. 22.1%), prolongation of TTP (5.2 *vs*. 2.9 months) and longer survival (median survival 18.5 *vs*. 15.8 months).

While the results of these studies illustrate the modest at best activity for the addition of antimetabolites to anthracycline/taxane and/or taxane-based therapy, results as related to TNBC should be interpreted with caution as only 40% of study populations were classified as triple negative. In addition the higher toxicity profile associated with doublet chemotherapy. So biomarker strategies to both enrich for responders and minimize toxicities associated with antimetabolites should be considered and incorporated into future neoadjuvant studies examining combination strategies^12^.

#### Neoadjuvant novel therapy in TNBC

##### Neoadjuvant antiangiogenic agents

Several investigators have sought to determine the benefit of targeting VEGF with bevacizumab in the neoadjuvant setting like the GeparQuinto study that was designed to determine the benefit of adding bevacizumab to anthracycline/taxane-based preoperative chemotherapy among women with HER2-negative breast cancer. Patients were randomized to receive 4 cycles of epirubicin/cyclophosphamide (EC) followed by 4 cycles of docetaxel (D) with or without bevacizumab. Approximately 35% of patients in both arms had TNBC. For the entire study cohort, there was no statistical significant difference in pCR between groups (15% EC → D and 17.5% EC → D plus bevacizumab). But in a predefined stratification by subtype, patients with TNBC had a significantly higher likelihood of pCR by the addition of bevacizumab compared to the other subtypes (OR =1.42)^37^.

In a subsequent analysis on a number of TNBC patients reported at ASCO 2011 annual meeting, pCR rates in both breast and lymph nodes were higher for patients who received EC → T plus bevacizumab compared to EC → D alone (36.4% *vs*. 28%)^38^.

An ongoing CALGB study 40603 evaluating both the addition to platinum and bevacizumab to standard anthracycline/taxane chemotherapy are eagerly awaited. And a large biomarker program is ongoing to try to identify subgroups within TNBC who achieve greater benefit from bevacizumab^12^.

##### Neoadjuvant ixabepilone

Ixabepilone is a novel semisynthetic antineoplastic agent derived from natural epothilones and their analogs. Similar to taxanes, ixabepilone stabilizes microtubules and causes cell cycle arrest and apoptosis. It has the advantage of bypassing the resistance mechanisms associated with drug efflux pumps and specific paclitaxel resistance associated with β-tubulin^39^.

The use of ixabepilone has been studied as a single agent in four distinct clinical trials. One of which was done in a number of women with inoperable breast cancer (of which 26% were TN) were treated with 4 cycles of single agent ixabepilone. pCR rates in the breast were 18% for the entire study population; 22% in ER negative/HER2 negative; 46.1% in ER negative/HER2 positive; 10.6% in ER positive/HER2 negative; and 20% in ER positive/HER2 positive. Gene expression studies from pretreatment core breast biopsies confirmed the inverse relationship between ER expression and ixabepilone sensitivity^40^.

An ongoing clinical trial evaluating differential responses to neoadjuvant paclitaxel versus ixabepilone following AC chemotherapy in the preoperative setting of locally advanced breast cancer including TNBC is eagerly awaited (NCT00455533). And PACS08 trial will evaluate responses of patients to neoadjuvant FEC100 followed by ixabepilone in TNBC^12^.

#### Adjuvant chemotherapy in TNBC

The importance of optimizing early-stage chemotherapy in TNBC is due to increased risk of recurrence within 3 years, increased risk of distant metastases and brain metastases with rapid progression from distant recurrence to death and non-validated targets for therapy^41^.

##### Anthracyclines and cyclophosphamide

The benefit of anthracycline-based therapy in TNBC is supported by several neoadjuvant studies. Also The WSG 01 trial supports the benefit from adjuvant anthracycline where it assigned patients with more than nine involved lymph nodes to receive either dose-dense conventional chemotherapy (i.e., 4·EC followed by 3 CMF q2w) or a rapidly cycled tandem high-dose regimen (i.e., 2·EC q2w followed by 2 epirubicin 90 cyclophosphamide 300 - thiotepa 400 q3w). In this study, young patients with TNBC and/or G_3_ tumors derived greater benefit from the rapidly cycled tandem approach than from the dose-dense conventional regimen. The high-dose approach lead to 5-year event-free survival rates as high as 71% in patients with TNBC compared with only 26% in TNBC patients treated by conventional dose-dense chemotherapy^14^.

Whereas patients with HER-2-overexpressing and/or topoisomerase-IIa-abnormal breast cancers have repeatedly been indicated to derive the most pronounced benefit from anthracycline-containing chemotherapy results, the efficacy of anthracycline-based regimens in patients with TNBC remain controversial despite the high RR to anthracycline as supported by several neoadjuvant chemotherapeutic studies^15^.

The controversy arise from the fact that TNBC is a heterogeneous disease and it remains unclear with regard to anthracycline sensitivity whether BRCA1 associated TNBC is functionally similar to sporadic TNBC as many provocative studies suggest that BRCA1 associated TNBC may be less sensitive to anthracycline-based therapy^14^^,^^16^.

Among a number of TN patients who received 6 cycles of FEC100, 22% BRCA1 carriers were identified. The pCR rate for the triple negative BRCA1 carriers was 17% compared with 42% in the rest of sporadic triple-negative non-carriers. However, other studies come to different conclusions and suggest that BRCA1/2 mutation carriers do indeed have high pCR rates to anthracyclines^42^.

Also, Berrada *et al*.^43^ reported on patients with breast cancer receiving six cycles of CEF, a subgroup identified as p53^+^/BLBC had derived particular benefit from this chemotherapy.

Also findings from a pooled subgroup analysis of eight adjuvant anthracycline trials assessing outcomes by HER2 status indicated a lack of benefit for anthracyclines in HER 2-negative disease^16^.

Moreover, subgroup analyses of individual trials have indicated mixed results for anthracycline-based therapy in TNBC subpopulations; some studies indicate a favorable effect in basal-like or TN tumors, while others indicate a lack of benefit^43^^,^^44^.

##### Adjuvant cyclophosphamide, methotrexate, and flurouracil (CMF)

Although most studies support the benefit of anthracycline based regimens in TN, analysis from the MA5 study comparing adjuvant cyclophosphamide, epirubicin, and fluoruracil (CEF) to CMF showed an improvement in 5-year overall survival in the CMF arm for TNBC (71% *vs*. 51%), whereas the CEF arm was superior in all other subgroups^44^.

The results of this study challenge the role of anthracyclines in adjuvant therapy for TNBC/BLBC but additional data will be needed for final clarification of this issue^14^.

##### Adjuvant taxanes

The first trial that established the benefit of paclitaxel added to AC in TNBC was CALGB 9344/INT1048. This trial randomized patients with node positive operable breast cancer to receive three different doxorubicin doses followed by further therapy with or without 4 cycles of paclitaxel every 3 weeks. The addition of paclitaxel resulted in a 17% reduction in the risk of recurrence and 18% reduction in the risk of death with an improvement in 5-year DFS and OS from 65% to 70% and 77% to 80%, respectively. Paclitaxel was associated with improvements in DFS in the HER2 positive patients regardless of hormone receptor status, whereas in HER2 negative patients, benefit was only seen in the hormone receptor negative group i.e., TNBC, and this suggests that the TN subset of breast cancer derives substantial benefit from the addition of paclitaxel in the adjuvant setting supporting the conclusion that taxanes are important in TNBC^45^.

Another large trial on a number of patients randomized to receive adjuvant doxorubicin and cyclophosphamide followed by docetaxel or paclitaxel given weekly or once every 3 weeks demonstrated an overall improvement in 5-year DFS and OS of 27% and 32%. In the triple negative subgroup the benefit of weekly paclitaxel was 37% over the 3-week regimen. Thus, not only is paclitaxel effective in this setting, but the weekly regimen is more active than the less frequent 3-week regimen^46^.

The benefit of taxanes in adjuvant therapy of TNBC has been realized over the past few years. However sensitivity of BRCA1-mutated cells to taxanes remains controversial as *in vitro* evidence on BRCA1 genotype-specific sensitivity to commonly used chemotherapy drugs indicate that BRCA1 mutations may confer resistance against taxanes^47^^,^^48^.

The NSABP B28 trial comparing doxorubicin and cyclophosphamide with or without four cycles of paclitaxel found no statistically significant difference in the relative risk of recurrence and overall survival based on hormone receptor status^49^.

Despite these confusing data to date, there is no convincing clinical evidence regarding a decreased sensitivity to taxanes in TNBC *vs*. non-TNBC.

##### Adjuvant capecitabine

The efficacy of capecitabine has not been prospectively studied in TNBC and there remains relatively scant data on its activity in this group. However, several observations can be made from retrospective subgroup analyses and several trials are underway to evaluate capecitabine in TNBC. In CALGB49907 study, standard adjuvant chemotherapy (either CMF or AC) was compared to capecitabine in women over age 65 to determine non inferiority and after a number of patients were enrolled in this study, and it was found that capecitabine was inferior to standard chemotherapy^50^.

But findings from subgroup analysis of two large randomized adjuvant capecitabine trials indicate that the addition of capecitabine to anthracyclines and taxanes may be particularly effective in TNBC populations^22^^,^^51^.

#### Palliative chemotherapy in metastatic patients

There is a predilection for visceral metastasis, including lung, liver, and notably brain. Approximately 15% of TNBC patients develop brain metastasis. In addition to having a short DFS, TNBC are aggressive in the metastatic setting, significantly due to shortened overall survival^3^. Historically, treatment standards for metastatic breast cancer (MBC) have included re-challenging with taxanes if the disease-free interval has been sufficiently long (usually >12 months) and the use of single agent capecitabine or vinorelbine for those who relapse shortly (<6-12 months) after completion of adjuvant taxane treatment. However, there are no current standards for TNBC therapy in the metastatic setting^52^.

The decision as to which type of chemotherapy/regimen should be given to patients with metastatic TNBC as first-line chemotherapy should be based on the individual (i.e., performance status, biological age, and co-morbidities) and their specific disease characteristics (i.e., tumor burden and disease-free interval), prior treatments received in the adjuvant setting, as well as patient preference^53^.

##### Taxanes in metastatic TNBC

Despite most of the recommendation indicated the use of taxanes in the metastatic setting, several trials suggest a lack of specific benefit of taxanes in TNBC over other subtypes. In the CALGB9342 trial, which evaluated three different doses of paclitaxel for MBC, there was no statistically significant difference in RR or time to treatment failure between TNBC and hormone receptor positive tumors. However, the overall survival was significantly worse for the TNBC compared to hormone receptor positive^47^.

The BRCA function may play a role in taxanes sensitivity. Preclinical data demonstrate that intact BRCA1 function contributes to anti-microtubule agent sensitivity^17^.

Also among a number of BRCA1 carriers identified in a registry of patients, only 40% who received docetaxel and doxorubicin had a complete or partial response compared to 100% who received non-taxane, DNA damaging regimens^54^.

##### Platinum agents in metastatic TNBC

In addition to the evaluation of platinum in the preoperative setting, studies in the metastatic setting further support that platinum may be active in metastatic TNBC. The TBCRC009 trial is done on a number of patients with metastatic TNBC. The patients were assigned to receive either cisplatin 75 mg/m^2^ IV every 3 weeks or carboplatin AUC of 6 every 3 weeks. Median PFS was 89 days. Thirty-three percent of patients had a PFS of less than 6 weeks and another 33% had a PFS longer than 6 months. Among responders, a median PFS was 242 days^55^.

The large randomized phase III TNBC trial (TNT) in the UK is underway comparing carboplatin with docetaxel for metastatic TNBC. The primary endpoint of the trial is RR. Patients may receive up to 6 cycles of treatment (carboplatin AUC 6 q3w-docetaxel 100 mg/m^2^) and will crossover to the other arm either at progression. The TNT study is designed to detect a 15% improvement in response to carboplatin compared to docetaxel. This trial will answer a critical question in the management of TNBC to help define how platinum should be utilized in metastatic disease^56^.

##### Capecitabine ± biological therapy in metastatic TNBC

O’Shaughnessy *et al*.^57^ reported that adding capecitabine to docetaxel provided benefit in metastatic BC whatever the ER status. Similar observations were made in the trials that evaluated the combination gemcitabine/vinorelbine and gemcitabine/paclitaxel^58^^,^^59^.

A phase II study found capecitabine with bevacizumab nearly doubled the RR in ER^+^ patients compared to triple negative patients (47% *vs*. 27%) with a similar difference in time to progression (8.9 *vs*. 4.0 months) and overall survival (>16.6 *vs*. 7.5 months). Results from such studies have caused some to conclude that capecitabine may be less effective in TNBC. However, additional data are needed before concluding that capecitabine has limited activity in TNBC^50^.

##### Novel therapy in metastatic TNBC

It has been shown that basal-like breast carcinomas frequently harbor defects in DNA double strand break repair through homologous recombination such as BRCA1 dysfunction. The DNA-repair defects characteristic of BRCA1-deficient cells confer sensitivity to poly (ADP-ribose) polymerase 1 (PARP1) inhibition^60^. The PARP1 gene encodes a chromatin-associated enzyme that modifies various nuclear proteins. This gene is involved in the molecular events leading to cell recovery from DNA damage. When PARP1 is inhibited, breaks in double-strand DNA accumulate and under normal conditions, would be repaired via homologous recombination. Both BRCA1 and BRCA2 are required for the homologous recombination pathway to function properly. Therefore, cells deficient in either BRCA1 or BRCA2 are sensitive to PARP1 inhibition, resulting in cell death and apoptosis. So, inhibition of the PARP pathway should benefit patients with BRCA-associated malignancies^61^.

Several PARP1 inhibitors hold promise in TNBC. The results of a randomized phase II study with BSI-201 (a PARP inhibitor) showed benefit in patients with TNBC who had two or fewer previous lines of chemotherapy. When BSI-201 was combined with gemcitabine and carboplatin, the clinical benefit rate improved to 62% compared with 21% in the gemcitabine and carboplatin alone arm (*P*<0.0002). Clinical benefit rate is defined as complete response plus partial response plus stable disease for at least 6 months. In addition, the overall response rate (ORR) was notably improved in the BSI-201 arm at 48% compared with the control arm at 16%. Progression-free survival (PFS) was improved to 6.9 months in the BSI-201 arm *vs*. 3.3 months in the gemcitabine and carboplatin alone arm^62^.

Iniparib belongs to a class of drugs called PARP inhibitors. Iniparib is currently in clinical trials for the treatment of certain kinds of breast cancer, included TNBC^63^. Many researches under investigation evaluating the response of iniparib in metastatic TNBC either as single agent or in combination with taxanes and other chemotherapeutic regimen as iniparib carries a promising and a challenging effect in management of TNBC^11^.

Multiple randomized trials have demonstrated improvements in PFS with the addition of bevacizumab to chemotherapy in first-line disease TN^64^^-^^66^.

Another novel mitotic inhibitor currently being studied for the treatment of breast cancer is eribulin. A phase III trial compared eribulin against several investigator-chosen regimens for the treatment of women with refractory MBC. An improved survival in favour of those women taking eribulin was demonstrated (median OS was 13.1 *vs*. 10.7; HR =0.81; 95% CI, 0.66-0.99). Of the patients enrolled in this trial, 20% had TNBC^67^.

ABI-007 (nab-paclitaxel) (abraxane), a novel nanoparticle albumin bound (nab) formulation of paclitaxel has shown improved PFS in 1^st^ and 2^nd^ line treatment of metastatic TNBC either alone or in combination with other chemotherapy^68^. In a phase I trial, the lower toxicities of abraxane allowed the administration of 70% higher dose than the approved dose of taxol (300 *vs*. 175 mg/m^2^, q3w) over shorter infusion time (30 min *vs*. 3 h), without the need for corticosteroid premedication^69^.

In a randomised phase III study in patients with MBC, compared with taxol at 175 mg/m^2^ q3w, Abraxane administered at 260 mg/m^2^ q3w had statistically significantly higher RRs, longer time to tumor progression, and increased survival in the subset of patients receiving second-line or greater treatment. The incidence of grade 4 neutropenia and hypersensitivity reactions with Abraxane were significantly lower than in the taxol group. Incidence of grade 3 neuropathy was higher for Abraxane due to higher dosage but was easily managed and improved quickly^70^.

In patients with metastatic TNBC resistant to anthracycline based or taxane-based chemotherapy, Rugo *et al*.^52^ reported improved PFS (4.1 *vs*. 2.1 months) and ORR (27% *vs*. 9%) for the novel microtubule-stabilizing agent (ixabepilone) in combination with capecitabine compared with capecitabine alone.

### Target therapy in TNBC

#### Mammalian target of rapamycin (mTOR) inhibitors

mTOR is one of the intracellular kinases. mTOR inhibitors have been shown to improve outcome in several cancer types including renal cancer. TNBC presents a high frequency of PTEN (phosphatase and tensin homolog) loss and mTOR activation. There is therefore a rationale to develop mTOR inhibition in patients with TNBC that show PTEN loss^71^. Interestingly, several reports say that mTOR activation could lead to cisplatin resistance, a phenomenon reversible by everolimus which is mTOR inhibitor^72^.

Beuvink *et al*.^72^ reported that adding everolimus to cisplatin could increase by 5-fold the loss of viability *in vitro*. These data suggest that there is a rationale to combine cisplatin and mTOR inhibitors in patients with TNBC.

FGFR inhibitors represent a new drug family. These drugs are either FGFR specific or target FGFR as part of their tyrosine kinase panel in addition to VEGFR inhibition. At least four compounds are currently under clinical trials on TNBC^11^.

EGFR signaling has been inhibited in other cancer types with clinical success either by using EGFR directed antibodies such as cetuximab or the inhibitors of receptor phosphorylation as gefitinib and erlotinib^73^. Cetuximab (erbitux) is a chimeric monoclonal antibody targeting EGFR, elicits little response to single-agent therapy in the setting of advanced TNBC^74^.

The activity of cetuximab in the treatment of TNBC alone or in combination with carboplatin is currently being investigated in the metastatic setting. In a phase II trial evaluating the combination of cetuximab and carboplatin with AUC=2, weekly for 3 of 4 weeks reported a RR of 18% and overall clinical benefit rate of 27% among a number of patients with advanced pretreated TNBC. Time to progression was 2 months and overall survival was 12 months which reflects the aggressive nature of this disease^75^ (**Table 1**).

**Table 1:** Cisplatin in triple negative breast cancer

Author	*n*	Setting	Regimen	Efficacy
Garber *et al*.^31^	28	Neoadjuvant	Cisplatin	pCR rate: 22%
Ezzat *et al*.^32^	126	Neoadjuvant	Cisplatin/ paclitaxel	pCR rate: 28%
Frasci *et al*.^33^	74	Neoadjuvant	Cisplatin/epirubicin/paclitaxel	pCR rate: 65%
Byrski *et al*.^55^	20	Metastatic	Cisplatin or carboplatin	cCR rate: 45%
Kilburn *et al*.^56^	400	Metastatic	Carboplatin or docetaxel	15% improvement in carboplatin
Carey *et al*.^75^	102	Metastatic	Carboplatin + cetuximab	RR: 18%

## Conclusion

TNBC represents a challenge for patients and clinicians due to its poorer prognosis and fewer treatment options, with a lack of targeted use of therapies which are reflected with high mortality in comparison to other breast cancer subtypes. As regard surgery in TNBC and despite being more aggressive disease, surgical decision making likely rests on more traditional clinicopathological variables (like patient age, tumor size, and tumor grade) and patient preference.

As regard radiotherapy in TNBC, it is given traditionally as indicated in other breast cancer subtypes following mastectomy or CBS, but there is still some controversy on this issue. The controversy arises from the fact that TNBCs are rapidly growing and locally aggressive cancers that may represent a limit to the general principle saying that breast-conserving surgery followed by radiation therapy in early stage (T_1-2_N_0_) is equivalent to mastectomy. Also the general consensus that postmastectomy radiation therapy is not indicated for patients with node-negative tumors less than 5 cm in diameter should not oversimplified in TN tumors.

The therapeutic strategies for the management of TNBC are targeting DNA repair complex like (platinum compounds and taxanes), p53 like (taxanes), cell proliferation like (anthracycline containing regimen) and targeted therapy. Neoadjuvant chemotherapy studies have consistently reported higher RRs in TNBC than non-TNBC and pCR has been shown to predict improved long-term outcomes for TNBC.

The specific adjuvant regimens that may be most effective for TNBC are still being to be determined. Numerous large randomized trials have established the benefit of adjuvant anthracyclines and taxanes in breast cancer.

All options are proposed in first-line treatment but the majority of recommendation indicated taxanes for first-line therapy while recommendations for second-line therapy were more commonly single agent capecitabine or combination of capecitabine and vinorelbine, or gemcitabine and vinorelbine or a platinum-based regimen.

The most frequently recommended platinum-based regimens for first-line therapy and second-line is cisplatin plus gemcitabine, carboplatin plus paclitaxel and carboplatin plus gemcitabine.

Several targeted therapies are being successfully developed like Poly (ADP-ribose) polymerase-1 (PARP-1), which is a nuclear DNA-binding enzyme activated by DNA strand breaks and has a key role in the signaling of DNA single-strand breaks as part of the repair process. Initial exciting data suggesting that iniparib improved outcome in patients with TNBC in combination with chemotherapy have not been confirmed in phase III studies, although there are clearly patients who benefit from this agent.

Several other targeted agents are being developed in the setting of managing TNBC including epidermal growth factor receptor (EGFR), FGFR2, VEGF, and mTOR.
